# Corrosion, Wear, and Fretting Corrosion Properties of Cr/CrN and Mo/MoN Multilayer Coatings with Biomedical Potential

**DOI:** 10.3390/molecules30234640

**Published:** 2025-12-03

**Authors:** Lin Chen, Bingyan Chen, Boxing Han, Heng Liu, Tianyi Zhang, Baojun Dong

**Affiliations:** 1 College of Safety and Ocean Engineering, China University of Petroleum, Beijing 102249, China; 2 Key Laboratory of Oil and Gas Safety and Emergency Technology, Ministry of Emergency Management, Beijing 102249, China; 3 School of Energy Power and Mechanical Engineering, North China Electric Power University, Beijing 102206, China; 4 College of Engineering, China University of Petroleum-Beijing at Karamay, Karamay 834000, China

**Keywords:** nitride hard coatings, magnetron sputtering, corrosion, wear, tribocorrosion

## Abstract

In this study, Cr/CrN and Mo/MoN alternating multilayer coatings with pure metal interlayers were deposited on 316 L stainless steel substrates via physical vapor deposition to systematically investigate the corrosion resistance, wear resistance, and tribocorrosion behavior of the multilayer coating systems in physiological saline environments. Microstructural characterization revealed that the CrN layer consists of mixed CrN and Cr_2_N phases, whereas the MoN layer exhibits a highly densified microstructure along with the presence of MoO_2_ phase, which collectively contribute to the superior corrosion resistance of the Mo/MoN coating. Furthermore, compared to the CrN layer, the MoN layer demonstrates enhanced nanomechanical properties and improved resistance to crack initiation, due to the greater hardness and higher H/E and H^3^/E^2^ values. Consequently, the Mo/MoN coating exhibits significantly better wear and tribocorrosion performance than its CrN counterpart. This work provides a theoretical foundation for the design of tribocorrosion-resistant hard coatings for artificial joint materials.

## 1. Introduction

Fretting wear and fretting corrosion occur at the fixation interface of artificial hip joints, accelerating the release of metal ions and leading to the failure of joint replacement surgery [1,2]. The deposition of surface protective coatings can shield against physical damage and chemical erosion, thereby extending its service life. Transition metal nitrides (TMNs) are formed from transition metals (such as Ti, Cr and Zr) and nitrogen [3,4,5,6]. TMNs coatings, as hard ceramic coatings, have high hardness [7,8], excellent corrosion resistance, and wear resistance [9,10]. These advantages make them one of the core materials for tribological [11,12] and corrosion-resistant applications. Physical vapor deposition (PVD) [13] and chemical vapor deposition (CVD) [14] are commonly used methods for depositing hard coatings and have been widely applied. PVD coatings offer tighter bonding with the substrate [15] and have advantages such as low cost and superior wear resistance. The multilayer design of TMN coatings can improve the mechanical properties and oxidation/wear resistance of the coatings [16,17,18,19]. Passivated metallic materials, such as stainless steel, are widely used in the medical implants (e.g., bone plates, screws, and stents) [20], petrochemical industry, marine industry, and automotive industry [21,22] due to their mechanical properties and ease of processing.

The chemical inertness, bioactivity, biocompatibility, and corrosion resistance of bioceramics render them highly suitable for surface functionalization of metallic implants. A representative example is the application of TiN coatings in dental implants [23,24], which is attributed to their excellent biological properties—such as mitigating the release of cobalt-chromium-molybdenum (CoCrMo) ions. An in vitro experiment showed that the TiN and TiCrN coatings were antibacterial (>50%), highly biocompatible, and noncytotoxic. In vitro experiments demonstrate that TiN and TiCrN coatings exhibit antibacterial properties (>50%), high biocompatibility, and no cytotoxicity [25]. In the field of coatings, chromium nitride (CrN) has been extensively studied, but molybdenum nitride (MoN) coatings are still in the research stage. It has been reported that MoN coatings exhibit a multi-phase structure [26,27] and possess high hardness, excellent adhesion to the substrate, and superior tribological properties [18,28,29,30]. These characteristics are attributed to the regulatory effect of the oxide layer/film formed at the MoN interface on friction behavior [17,31]. Furthermore, the adoption of multilayer architectures (such as alternating soft/hard phase structures) effectively mitigates internal stresses and brittleness [26,32,33,34,35,36]. However, the corrosion and tribocorrosion behavior of multilayer Cr/CrN and Mo/MoN coatings in a biomimetic environment remains unclear, which restricts their application in the field of biomedical materials.

In this study, multi-layer Cr/CrN and Mo/MoN coatings containing a soft metal intermediate layer were prepared on a 316 L stainless steel substrate using magnetron sputtering technology, and the coating surface morphology, phase structure, and elemental composition were characterized. The nano-mechanical properties of those coatings were analyzed using nanoindentation, and the corrosion resistance was analyzed using were evaluated by potentiodynamic polarization (PDP) and electrochemical impedance spectroscopy (EIS). In addition, through comparative analysis of Cr/CrN and Mo/MoN coatings, the differences in wear and tribocorrosion resistance between the two coatings and their microscopic mechanisms were elucidated.

## 2. Results

### 2.1. Coating Characterization

**Figure 1:**
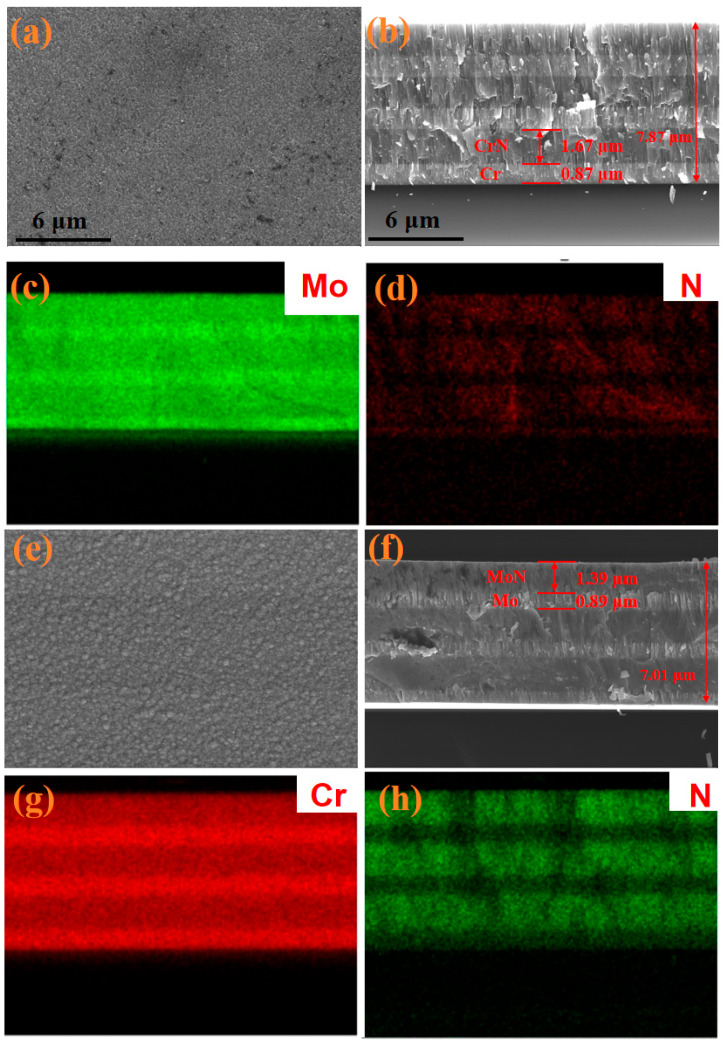
Microscopic morphology and distribution of element of the coatings: (**a**) surface and (**b**) cross-sectional morphology of the MoN coating; (**c**,**d**) distribution of elements; (**e**) surface and (**f**) cross-sectional morphology of the CrN coating; (**g**,**h**) distribution of elements.

The surface morphology and cross-sectional information of the prepared multilayer Cr/CrN and Mo/MoN coatings are shown in Figure 1. Both coatings were deposited using a cyclic multilayer deposition scheme, with a total of three cycles. Energy dispersive spectroscopy (EDS) clearly revealed the elemental distribution in the cross-section of the coatings (Figure 1c,d,g,h), showing that the nitride layer and metal intermediate layer were alternately arranged, and the pure metal layer did not contain nitrogen. As shown in Figure 1b,f, the total thickness of the Cr/CrN coating is approximately 7.8 μm, with the thickness of the single metal layer being approximately 0.87 μm and the thickness of the single nitride layer being approximately 1.67 μm. The total thickness of the Mo/MoN coating is approximately 7 μm. The thicknesses of the single metal layer and the nitride layer are 0.89 μm and 1.4 μm, respectively. From the cross-sectional morphology, it can be seen that the CrN layer has a typical columnar crystal structure, and its density is obviously inferior to that of the MoN layer. There are no obvious crack defects on the surface of the two coatings (Figure 1a,e).

**Figure 2:**
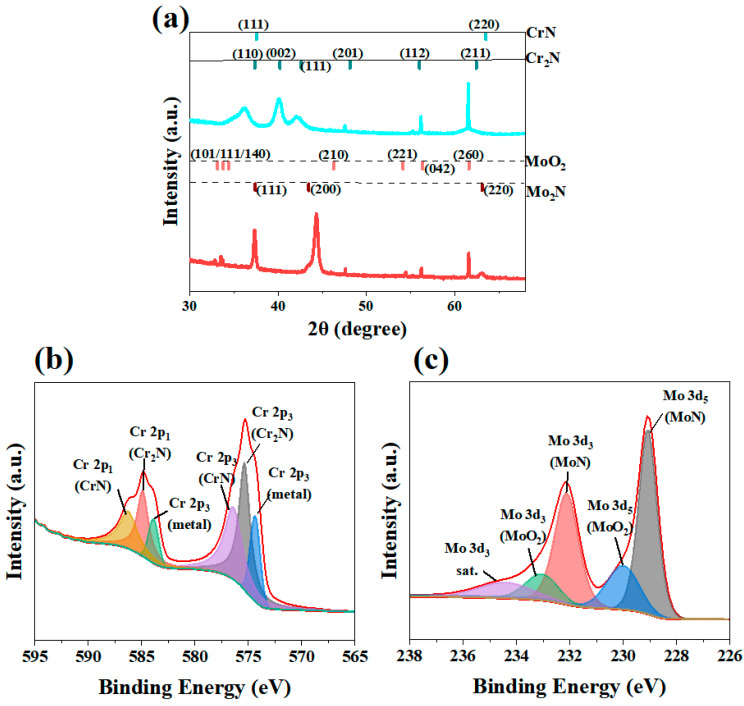
XRD and XPS spectra of the two coatings: (**a**) XRD patterns (Red indicates a MoN coating, while fluorescent colors denote a CrN coating), (**b**) XPS spectrum of Cr in the Cr/CrN coating, and (**c**) XPS spectrum of Mo in the Mo/MoN coating.

The phase composition and elemental chemical state of the coating were characterized by X-ray diffraction and X-ray photoelectron spectroscopy (Figure 2). The analysis results showed that in the multi-layer Cr/CrN coating, the nitride layer mainly consisted of CrN and Cr_2_N (PDF#35-0803, PDF#11-0065), and there was also a pure Cr metal intermediate layer, as shown in Figure 2a. In contrast, the nitride phase in the multilayer Mo/MoN coating is primarily composed of Mo_2_N (PDF#25-1366). Notably, the surface of the Mo/MoN coating undergoes oxidation in air, forming the MoO_2_ phase (PDF#05-0508). The shift in nitride peak positions is attributed to residual stresses. Under compressive stress, the lattice is compressed along certain directions, leading to a decrease in lattice spacing and a consequent shift in the diffraction peak to a higher 2θ angle. Conversely, under tensile stress [37,38], the lattice is elongated, resulting in an increased lattice spacing and a shift in the diffraction peak to a lower 2θ angle. In coatings fabricated by physical vapor deposition (PVD), the presence of internal stress is inevitable. Variations in tensile or compressive stresses across different regions of the coating likely account for the observed peak shifts of approximately 1–1.5°. The XPS analysis results (Figure 2c) further confirm that Cr, CrN (576.3 eV), and Cr_2_N (574.2 eV) phases were detected in the Cr/CrN coating, while Mo, Mo_2_N (228.8 eV), and MoO_2_ phases (232.0 eV) were detected in the Mo/MoN coating, consistent with the XRD analysis results.

**Figure 3:**
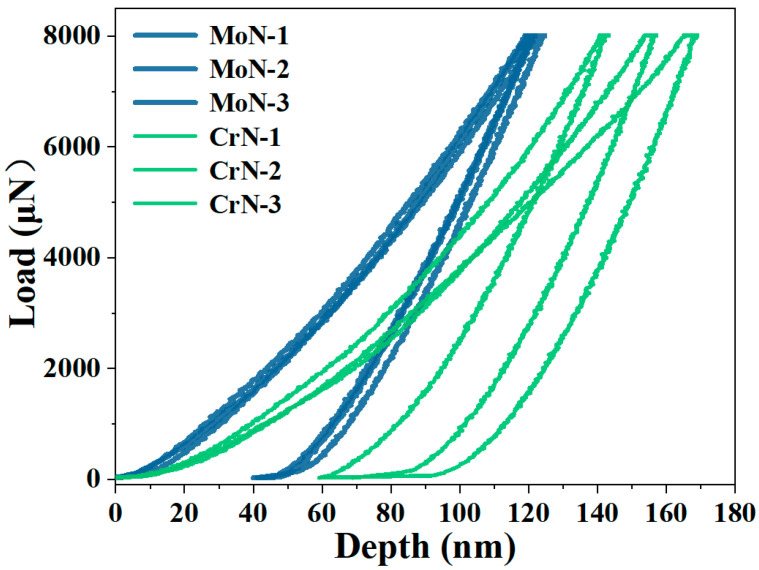
Nanoindentation curves of the CrN and MoN coatings.

The nanomechanical properties of both coatings were characterized by nanoindentation, with their load—displacement curves shown in Figure 3 and detailed mechanical parameters provided in Table 1. The results indicate that the hardness of the MoN coating reached 21.5 GPa, which is 50% greater than that of the CrN coating. The ratio of hardness (H) to Young’s modulus (E) can be used to evaluate the deformation resistance of coatings, and the MoN coating resulted in a significantly higher H/E value (0.1) and resistance to plastic deformation index H^3^/E^2^ (0.22) than the CrN coating did. These findings demonstrate that the MoN coating possesses superior deformation resistance and increased fracture toughness [39,40]. These differences in mechanical properties may influence the wear performance.

### 2.2. Corrosion Resistance Analysis

**Figure 4:**
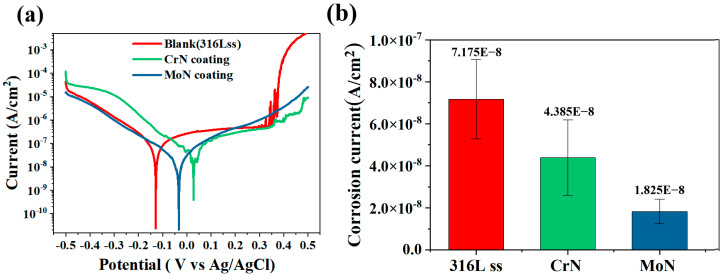
(**a**) Potentiodynamic polarization curves of the 316 L stainless steel substrate and the CrN and MoN coatings. (**b**) Statistical analysis of the corrosion current density.

Figure 4 displays the potentiodynamic polarization curves of the metallic substrate and two types of coatings. The results demonstrate that the corrosion resistance of 316 L stainless steel was significantly enhanced after coating deposition. The average corrosion current density of the 316 L stainless steel substrate was 7.18 × 10^−8^ A/cm^2^; after depositing the multilayer Cr/CrN coating, this value decreased to 4.39 × 10^−8^ A/cm^2^, whereas after depositing the Mo/MoN coating, the corrosion current density decreased by 4 times to 1.83 × 10^−8^ A/cm^2^. Compared with the Cr/CrN coating, the Mo/MoN coating exhibited superior corrosion resistance, which was closely related to the MoO_2_ oxide formed between the metallic Mo layer and the MoN layer. The pitting potential of 316 L stainless steel is 0.370 V (vs. Ag/AgCl), while the pitting potentials of the two coatings are both higher than 0.45 V (vs. Ag/AgCl). Compared with 316 L stainless steel, the coatings primarily influence the anodic region of the polarization curves, thereby enhancing the anti-pitting properties of 316 L stainless steel, as observed in the anodic region of Figure 4a.

**Figure 5:**
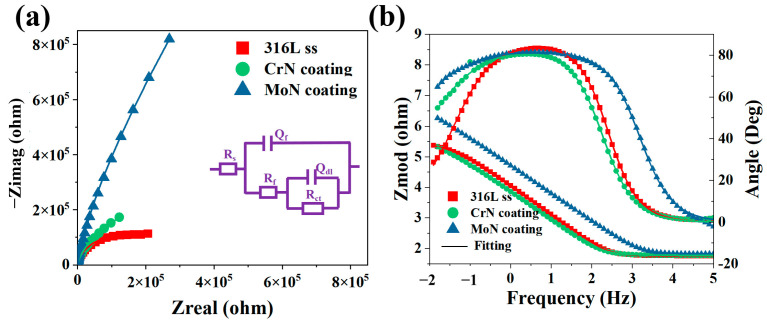
EIS spectra of three samples: (**a**) Nyquist plots and (**b**) Bode plots.

Figure 5 presents the electrochemical impedance spectroscopy (EIS) results of the metallic substrate and two coatings, with parameters fitted via the equivalent circuit models listed in Table 2. The data analysis indicated that after coating deposition, the corrosion resistance of the material improved by at least one order of magnitude compared with that of the substrate. Among them, the charge transfer resistance (Rct = 3.22 × 10^6^ Ω·cm^2^) of the Mo/MoN coating was approximately 3 times greater than that of the Cr/CrN coating, and a higher Rct value directly corresponds to a stronger corrosion protection capability. A comprehensive analysis of the potentiodynamic polarization and EIS results revealed that the Mo/MoN multilayer coating possessed optimal corrosion resistance; this is attributed to the dense oxide layer formed at the interlayer effectively inhibiting the penetration of corrosive media, thereby significantly enhancing the protective performance of the coating. The phase angle at middle frequencies (around 10^4^ Hz) can reflect the integrity of the coating layer [41]. The Mo/MoN coating showed a higher phase angle at this frequency range compared to the Cr/CrN coating, indicating a more intact and uniform coating structure. This structural integrity is crucial for preventing the ingress of corrosive substances and thus enhancing the overall corrosion protection. Moreover, the equivalent circuit model fitting results in Table 2 revealed that the Mo/MoN coating had a lower double-layer capacitance (Cdl) value compared to the Cr/CrN coating. A lower Cdl value typically implies a thicker and more compact passive film on the coating surface [42,43], which can effectively block the transfer of charge and ions between the coating and the corrosive environment, thereby improving the corrosion resistance. Overall, the comprehensive electrochemical analysis, including potentiodynamic polarization, EIS, and related parameter evaluations, clearly demonstrates that the Mo/MoN multilayer coating exhibits significantly better corrosion resistance than the Cr/CrN coating and the bare 316 L stainless steel substrate.

### 2.3. Fretting Wear

To investigate the wear resistance of two multilayer nitride coatings, fretting tests were conducted using a wear testing machine. Through systematic analysis of friction coefficient evolution, quantification of wear volume, microstructure of wear scars, and element distribution characteristics, a comprehensive evaluation was performed on the wear resistance and influencing mechanisms of the multilayer Cr/CrN coating and Mo/MoN coating.

**Figure 6:**
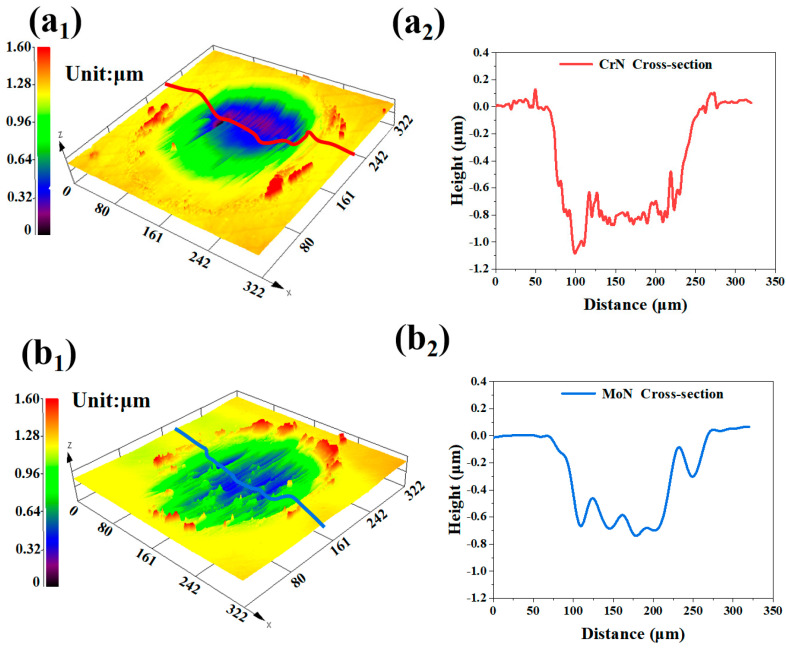
Three-dimensional morphological information of the wear tracks after 1 h of wear: (**a1**,**a2**) CrN coating and (**b1**,**b2**) MoN coating.

The three-dimensional morphology of the wear scars for both coatings after wear is shown in Figure 6, with detailed wear area and volume loss data provided in Table 3. The wear scar depth of the Cr/CrN coating reached 1.1 μm, whereas that of the Mo/MoN coating was only 0.7 μm. Furthermore, both the wear damage area and volume loss of the Cr/CrN coating were significantly greater than those of the Mo/MoN coating (Table 3). This demonstrates that the wear resistance of the Mo-based nitride layer surpasses that of the Cr-based nitride layer. Nanoindentation test results revealed that the Mo/MoN coating had increased nanohardness, Young’s modulus, and deformation resistance. These superior mechanical properties are directly related to its enhanced wear resistance.

**Figure 7:**
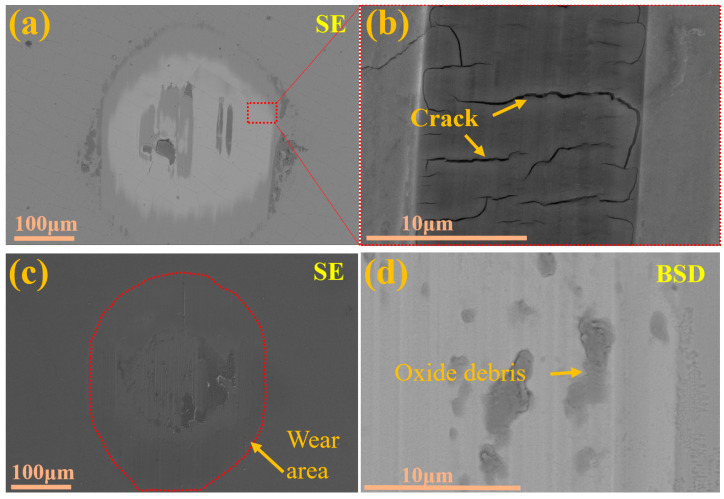
Morphology of the wear scar of the coating after 1 h of wear: (**a**,**b**) Cr/CrN, (**c**,**d**) Mo/MoN.

**Figure 8:**
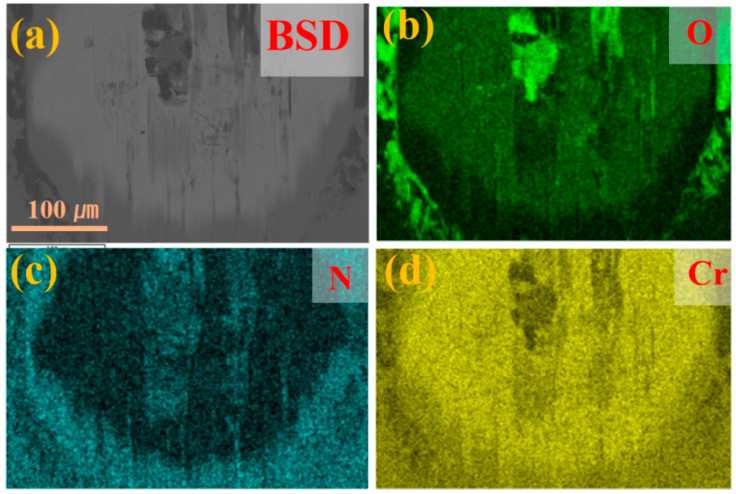
Elemental distribution within the wear track of the Cr/CrN coating: (**a**) Electron micrograph, (**b**–**d**) Distribution maps of O, N, and Cr elements.

**Figure 9:**
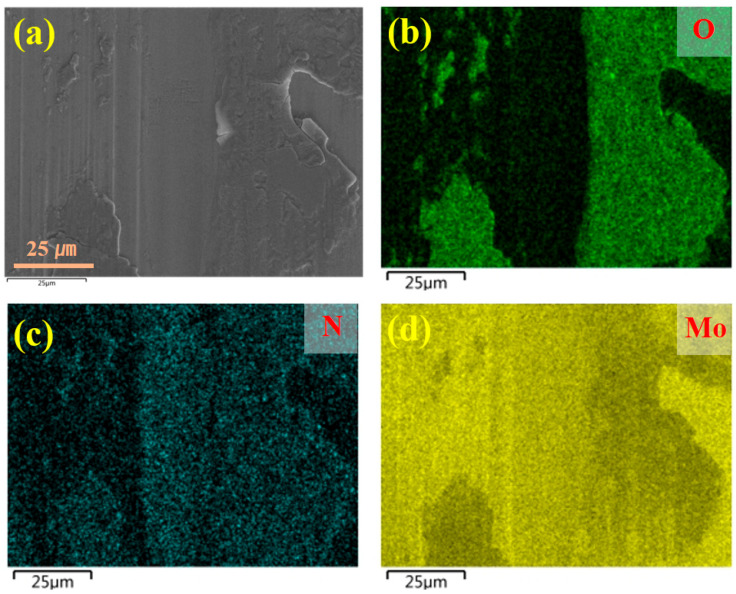
Elemental distribution within the wear track of the Mo/MoN coating: (**a**) Electron micrograph, (**b**–**d**) Distribution maps of O, N, and Cr elements.

Figure 7, Figure 8 and Figure 9 present the microstructure and energy-dispersive spectroscopy (EDS) analysis results of the Cr/CrN and Mo/MoN coating after 1 h of wear, respectively. As shown in Figure 7a,b, significant cracks were observed at the wear scar centre, indicating mechanical wear-induced cracking in the Cr/CrN layer. The EDS analysis results (Figure 8) revealed significant oxygen enrichment in the central region of the wear scar. The accumulation of wear debris at the wear scar edge and centre suggests the coexistence of abrasive wear mechanisms. Figure 7c,d display the wear scar morphology of the Mo/MoN coating, with substantial oxide accumulation at the wear scar centre. Distinct ploughing grooves are visible at the wear scar edge, whereas the central area exhibits smooth plastic deformation features, indicating that oxidative wear is the dominant mechanism for the Mo/MoN coating. The EDS spectra (Figure 9) verified the pronounced oxygen enrichment in this region. Notably, no cracks were observed in the Mo/MoN coating, which is consistent with its high deformation resistance, as revealed by nanoindentation.

Compared to the Mo/MoN coating, the Cr-based nitride layer exhibits lower hardness, Young’s modulus, and deformation resistance. Consequently, during wear testing, the Cr/CrN coating undergoes extensive brittle cracking and spalling, resulting in accelerated wear of the Cr-based nitride layer. Thus, the Mo/MoN coating demonstrates significantly superior wear resistance.

### 2.4. Fretting Corrosion

**Figure 10:**
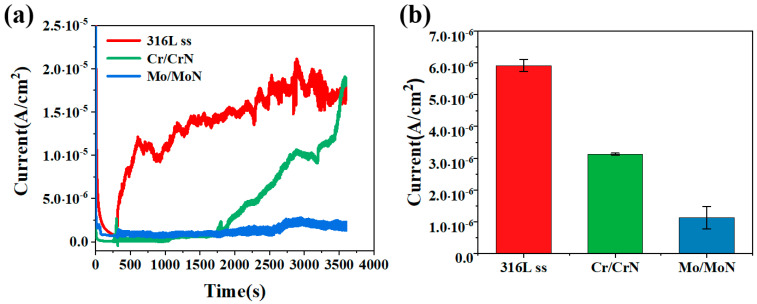
Potentiostatic fretting corrosion tests and corresponding statistics of the substrate and coatings: (**a**) current-time curves, (**b**) corrosion current statistics.

The fretting corrosion tests were conducted in a physiological saline solution containing 3% bovine serum albumin. Using a potentiostatic method, the fretting corrosion behaviors of the substrate and two coatings (Cr/CrN and Mo/MoN) were systematically evaluated by comparing corrosion current density and statistically analyzing material loss volume, as shown in Figure 10a. The results indicate that the average fretting corrosion current density of the 316 L stainless steel substrate was 5.9 μA/cm^2^. After deposition of the coatings, the fretting corrosion current density decreased significantly, with the Cr/CrN coating exhibiting a value of 3.1 μA/cm^2^, and the Mo/MoN coating further reducing it to 1.1 μA/cm^2^. Therefore, compared with the substrate, both nitride coatings demonstrated superior fretting corrosion resistance, with the Mo/MoN coating showing particularly outstanding protective performance. The fracture of the Cr/CrN coating during fretting corrosion exposed the metallic substrate, which led to a rapid increase in the corrosion current density, as shown in Figure 10a.

**Figure 11:**
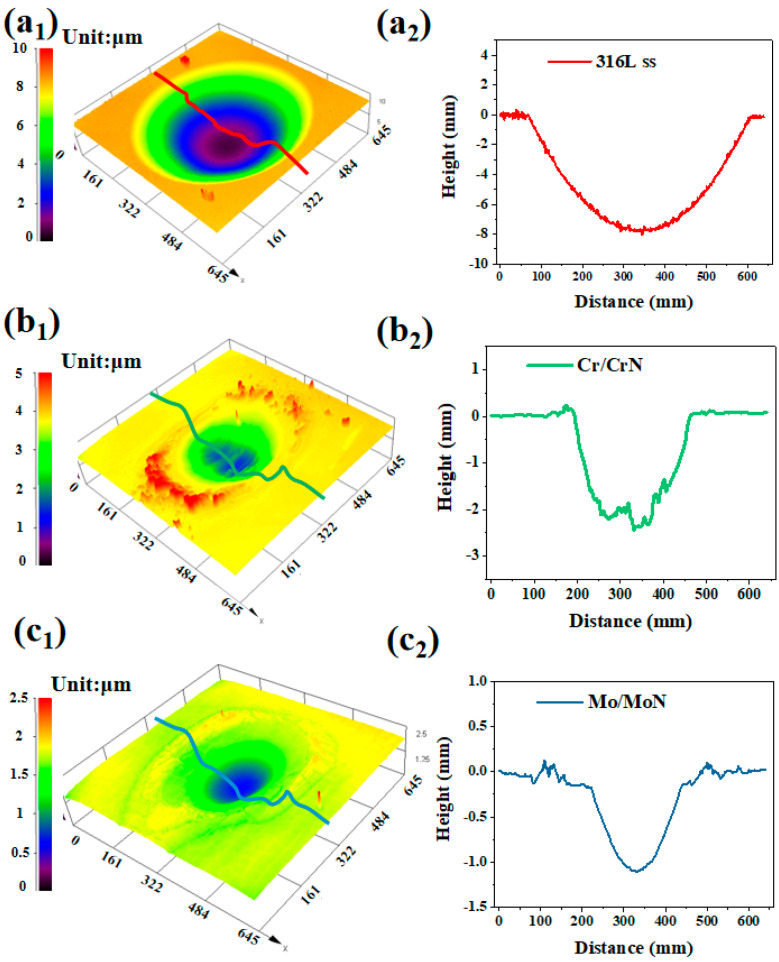
Three-dimensional topographies of the worn surfaces after fretting corrosion: (**a1,a2**) 316 L stainless steel, (**b1,b2**) Cr/CrN coating, (**c1,c2**) Mo/MoN coating.

The wear scars were further characterized using three-dimensional topography analysis, as shown in Figure 11, with the corresponding volume loss and wear scar depth statistics summarized in Table 4. The results indicate that 316 L stainless steel exhibited the highest accumulated volume loss, reaching 4.2 × 10^5^ μm^3^, along with a wear scar depth of 6.64 μm. In contrast, the volume losses of the Cr/CrN and Mo/MoN coatings were considerably lower, measuring only 4.3 × 10^4^ μm^3^ and 1.5 × 10^4^ μm^3^, respectively. Therefore, the ranking of fretting corrosion resistance is as follows: Mo/MoN > Cr/CrN > 316 L stainless steel.

## 3. Discussion

**Figure 12:**
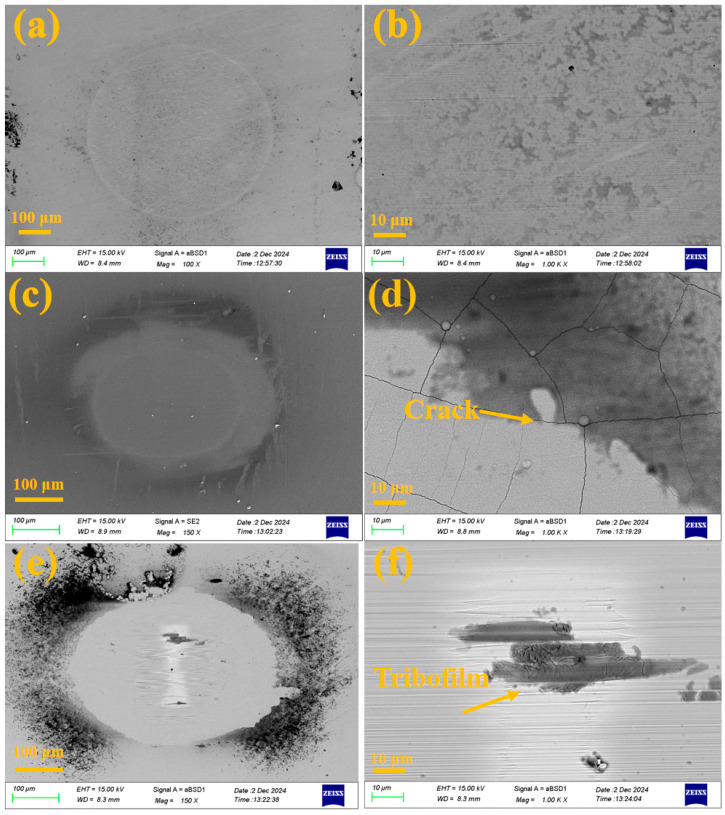
Characterization of the microscopic morphology of wear scars after fretting corrosion: (**a**,**b**) 316 L stainless steel, (**c**,**d**) Cr/CrN coating, (**e**,**f**) Mo/MoN coating.

Figure 12 displays the microscopic morphologies of the wear scars on the substrate and coating samples after fretting corrosion. As seen in Figure 12a,b, the wear scar of 316 L stainless steel is relatively smooth and contains a small amount of residual protein, which provides a certain lubricating effect.

**Figure 13:**
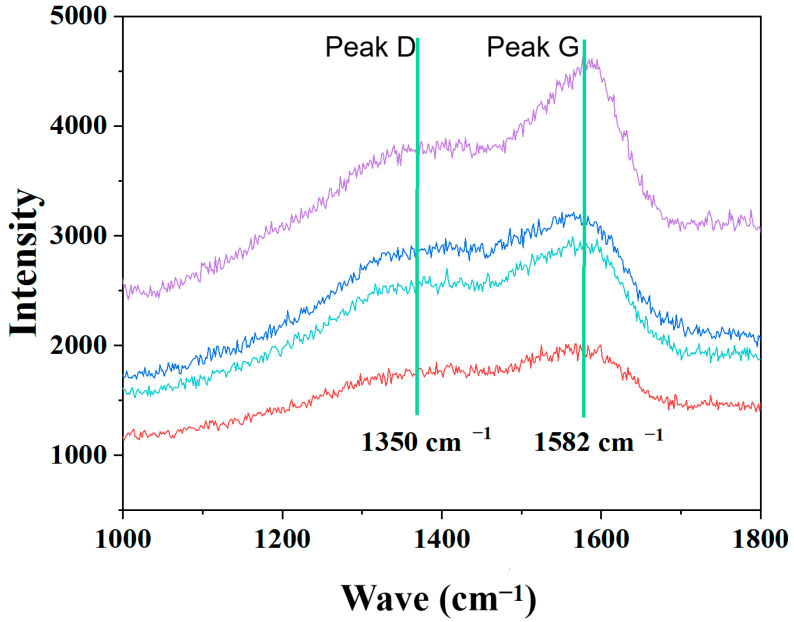
Raman Spectroscopic Characterization of MoN-Coated Friction Films (Different colors merely represent parallel data).

In comparison, Figure 12c,d reveal that the CrN coating surface contains numerous cracks, which can be attributed to its relatively poor toughness. Under mechanical loading, the coating readily fractured, exposing the underlying metal and leading to a rapid increase in corrosion current, as illustrated in Figure 10a. In contrast, no cracks were observed within the wear scar of the Mo coating, where a distinct tribofilm was formed. This behavior is attributed to the excellent mechanical properties of the Mo coating—a high H^3^/E^2^ ratio contributes to resistance against deformation and crack initiation. Moreover, the presence of molybdenum promotes the conversion of proteins in the solution into a tribofilm during the wear process, which effectively mitigates the wear progression. The tribofilm formed on the MoN coating was further characterized by Raman spectroscopy. As shown in Figure 13, the spectrum exhibits the characteristic D and G bands, indicating the presence of graphitic carbon within the tribofilm. The formation of this graphitic structure, which acts as a solid lubricant, contributes to a reduced friction coefficient and consequently enhances the wear resistance of the Mo/MoN coating. In contrast to TiN coatings, molybdenum metal exhibits a degree of catalytic activity, which is capable of enhancing tribochemical reactions and the subsequent formation of a tribofilm. This functionality underscores its potential biomedical relevance.

**Figure 14:**
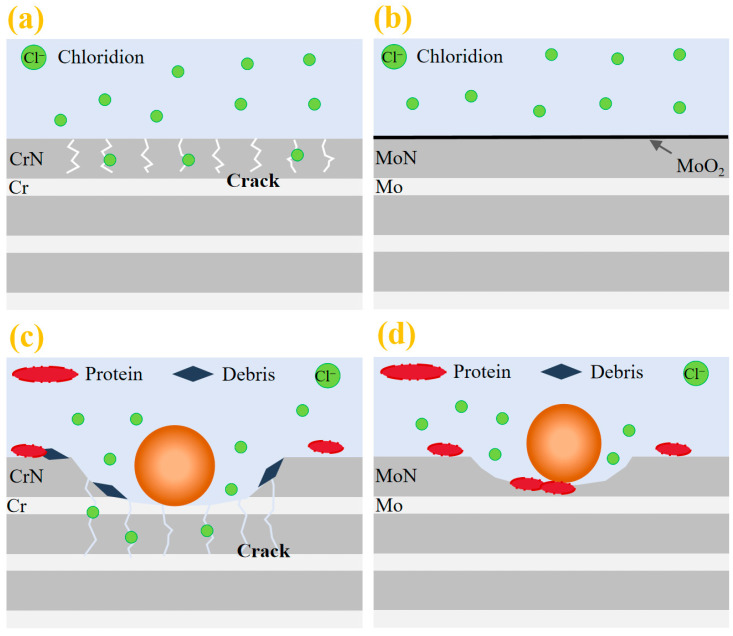
Corrosion and fretting corrosion behavior: (**a**) corrosion process of Cr/CrN coating and (**b**) Mo/MoN coating; (**c**) fretting corrosion of the Cr/CrN coating and (**d**) Mo/MoN coating.

In summary, it can be concluded that in terms of corrosion resistance, the compact microstructure of the Mo/MoN coating and its oxides effectively impede the ingress of corrosive ions, resulting in significantly superior corrosion resistance compared to the Cr/CrN coating, as illustrated in Figure 14a,b. Furthermore, the favorable mechanical properties of the Mo/MoN coating prevent cracking during wear and tribocorrosion processes. Additionally, the tribocorrosion products further hinder the penetration of corrosive ions, as shown in Figure 14c,d, thereby maintaining a consistently low tribocorrosion rate. Consequently, the Mo/MoN coating exhibits better fretting corrosion resistance than the Cr/CrN coating.

## 4. Materials and Methods

### 4.1. Coating Deposition

Medical-grade 316 L stainless steel (ASTM F138) was employed as the substrate material, and was cut into specimens measuring 10 mm× 10 mm× 2 mm. All substrates were progressively ground using SiC abrasive paper and subsequently mirror-polished. Prior to deposition, the substrates underwent ultrasonic cleaning in ethanol and acetone for 20 min each to eliminate surface contaminants and organic residues. High-purity (99.99%) chromium (Cr) and molybdenum (Mo) targets supplied by Yanbang New Materials Co., Ltd. (Beijing, China) were used as source materials. The coatings were deposited using a physical vapor deposition system (Beijing Tainuo Technology Co., Ltd., Beijing, China). The deposition process commenced by evacuating the chamber to a base pressure of 2 Pa at 250 °C. Substrate biasing was applied at −900 V to ignite glow discharge plasma, followed by 10-min in situ plasma cleaning to remove residual surface contaminants. Subsequently, under a working pressure of 0.2 Pa, the substrate bias was adjusted to −25 V with a deposition power of 100 W to deposit pure metallic interlayers. The metal deposition phase lasted 30 min, resulting in interlayer thicknesses ranging from 850 to 900 nm. This was followed by reactive deposition of nitride layers for 1.5 h using Ar/N_2_ gas flow ratios of 45 sccm:15 sccm. The alternating deposition cycle was repeated three times to construct the multilayer architecture. Finally, the coated specimens were vacuum-sealed in quartz tubes and subjected to post-deposition annealing at 500 °C for 6 h, followed by furnace cooling to ambient temperature.

### 4.2. Electrochemical Testing

Stainless steel substrate and coating samples were fixed in an electrochemical reaction cell and 10 mL of salt solution was added. The area of the substrate and coating exposed to the solution was 0.5 cm^2^. Corrosion performance testing was conducted using an electrochemical workstation (Gamry 1000, Warminster, PA, USA). The substrate and coating samples served as the working electrodes, with Ag/AgCl electrodes as the reference electrodes and platinum plate electrodes (10 × 10 mm) as the counter electrodes. Before the test begins, a cathodic potential of −0.8 V (vs. Ag/AgCl) was applied and maintained for 300 s to clean the oxides from the surface of the electrochemical sample. After completion, an open-circuit potential (OCP) test was performed and maintained for 30 min to ensure the stability of the sample surface. Subsequently, testing was conducted using the Potentiodynamic polarization method, with the potential ranging from −0.5 V to 0.5 V (vs. Ag/AgCl) and a scanning rate of 0.33 mV/s. In addition, electrochemical impedance spectroscopy (EIS) tests were conducted in the frequency range of 10^5^ Hz to 10^−2^ Hz with a perturbation amplitude of 10 mV (vs. OCP). Data analysis was performed using ZsimpWin software (3.30). The test solution was 0.9 wt% NaCl (mass fraction), and all tests were conducted at room temperature (25 °C). Constant potential testing was conducted during micro-corrosion, with the potential set at 0.1 V (vs. Ag/AgCl) for 3600 s in a 3 wt% bovine serum albumin solution.

### 4.3. Fretting

The coated sample was adhered to the organic glass plate by strong glue and fixed on a voice coil motor. The voice coil motor drives the coated sample to perform sinusoidal reciprocating motion, combined with a friction tester for fretting testing (UMT CERE-2, BRUKER, Billerica, MA, USA). For fretting wear testing and fretting corrosion testing, the frequency of the fretting wear was 1 Hz, and the reciprocating displacement was 100 µm. The friction pair was an alumina ceramic ball with a diameter of 9.562 mm that was fixed to a 3D mechanics sensor. The stepper motor with software controlled the sensor’s depressed load to be constant at 15 N and collected the tribological data of the fretting process. The fretting run time was 1 h; each test was repeated more than five times to obtain better reproducibility.

### 4.4. Morphology and Elemental Characterization

The morphology of the coating surface, cross-section, and wear marks was characterized using a scanning electron microscope (SEM, ZEISS, G300, Oberkochen, Germany), and the elemental distribution in the coating cross-section and wear marks was characterized using energy dispersive spectroscopy (EDS). The three-dimensional (3D) morphology, wear area, and volume of the wear marks were characterized using a laser scanning confocal microscope (OLS4100, Olympus, Tokyo, Japan). The elemental information of the coating and wear marks was analyzed using an X-ray photoelectron spectrometer (XPS, AXIS Ultra DLD, Manchester, UK) while the binding energies in the XPS spectra were calibrated against adventitious carbon (C 1 s 180 = 284.8 eV) with characteristic peaks fitted via XPS PEAK 4.1. The phase composition of the coating was characterized using an X-ray diffractometer (XRD, Rigaku Smartlab, Tokyo, Japan) using Cu Kα radiation over 10–90° 2θ at 2°/min. A nanoindenter (Hysitron TI 980, Bruker) was used to characterize the nanomechanical properties of the coating with the with a maximum load of 8000 μN during testing

## 5. Conclusions

In this study, multilayer coatings with potential for biomedical applications were fabricated using magnetron sputtering, and their physicochemical properties—including microstructure, phase composition, chemical states of elements, and mechanical properties—were systematically characterized. The corrosion resistance, wear resistance, and fretting corrosion resistance of Cr/CrN and Mo/MoN coatings were comparatively investigated, leading to the following conclusions:

(1) Phase composition analysis revealed that CrN and Cr_2_N are the dominant phases in the chromium-based nitride coatings, whereas Mo_2_N and Mo_2_O are the primary phases in the molybdenum-based system. In terms of mechanical properties, the Mo/MoN coatings exhibited superior performance compared to the Cr/CrN coatings, particularly in hardness, Young’s modulus, and resistance to deformation.
(2) The Mo/MoN coatings demonstrated significantly better corrosion resistance than the Cr/CrN coatings, which can be attributed to two main factors: the barrier effect of the MoO_2_ oxide layer against the penetration of corrosive species, and the inherently denser microstructure of the Mo-based coating. Accordingly, the corrosion resistance ranking was established as: Mo/MoN > Cr/CrN > 316 L substrate.
(3) Owing to their higher hardness and excellent deformation resistance, the molybdenum-based nitride coatings exhibited better wear resistance than the chromium-based nitride coatings. During fretting corrosion tests, no cracking was observed in the Mo/MoN coatings, indicating their high resistance to deformation-induced cracking. Furthermore, the combination of superior corrosion resistance and greater mechanical strength resulted in better overall fretting corrosion performance of the Mo/MoN coatings compared to the Cr/CrN system.

The novel MoN coating demonstrates promising potential for biomedical applications. However, its biocompatibility, particularly the biological response to wear debris such as interactions with macrophages and the potential to trigger adverse reactions (e.g., inflammatory response), requires further investigation. This represents a limitation of the present study, as such biocompatibility assessments were not conducted.

## Figures and Tables

**Table 1:** Nanomechanical properties of the CrN and MoN coatings.

Coating	Hardness (GPa)	Elastic Modulus (GPa)	H/E	H^3^/E^2^
CrN	13.5 ± 3.1	186.1 ± 11.4	0.07	0.07
MoN	21.5 ± 2.1	214.6 ± 10.6	0.10	0.22

**Table 2:** Fitting parameters of the electrochemical impedance spectra.

Sample	*Rs* (Ωcm^−2^)	*CPEf* (Ω^−5^ cm^−2^s^n^)	*n*_1_	*Rf* (Ωcm^−2^)	*CPEdl* (Ω^−5^ cm^−2^s^n^)	*n*_2_	Rct (Ωcm^−2^)
316 L ss	58.13	1.47	0.96	-	-	-	9.67 × 10^4^
CrN	60.27	2.49	0.93	1.08 × 10^5^	72.99	0.43	1.08 × 10^6^
MoN	66.96	36.68	0.91	3.40 × 10^6^	20.24	1	3.22 × 10^6^

**Table 3:** Statistics of the wear track area and volume loss after 1 h of wear.

Coating	Volume Loss (μm^3^)	Error (μm^3^)	Area (μm^2^)	Error (μm^2^)
CrN	17,693	77	48,862	459
MoN	12493	342	36079	107

**Table 4:** Statistical results of wear scar depth and volume loss after fretting corrosion.

	Volume (μm^3^)	Error (μm^3^)	Depth (μm)	Error (μm)
316 L ss	417,786	96,761	6.64	1.48
Cr/CrN	42,788	3121	2.38	0.21
Mo/MoN	15,324	175	1.05	0.02

## Data Availability

The original contributions presented in this study are included in the article. Further inquiries can be directed to the corresponding authors.
